# ROCK2, but not ROCK1 interacts with phosphorylated STAT3 and co-occupies TH17/TFH gene promoters in TH17-activated human T cells

**DOI:** 10.1038/s41598-018-35109-9

**Published:** 2018-11-09

**Authors:** Wei Chen, Melanie S. Nyuydzefe, Jonathan M. Weiss, Jingya Zhang, Samuel D. Waksal, Alexandra Zanin-Zhorov

**Affiliations:** 1 Kadmon Corporation, LLC, New York, NY 10016 USA; 2 Current Weill Cornell Medicine, New York, NY 10021 USA

## Abstract

Rho-associated coiled-coil kinase (ROCK)2 targeting down-regulates autoimmune responses in animal models and patients, however the underlying molecular mechanism is still an enigma. We report that ROCK2 binds phosphorylated-STAT3 and its kinase activity controls the formation of ROCK2/STAT3/JAK2 complex and optimal STAT3 phosphorylation in human CD4^+^ T cells during T helper 17 (TH17)-skewing. Moreover, chromatin-immunoprecipitation sequencing (ChIP-seq) analysis revealed that, genome-wide, about 70% of ROCK2 and STAT3 peaks overlapped and co-localized to several key genes controlling TH17 and T follicular helper (TFH) cell functions. Specifically, the co-occupancy of ROCK2 and STAT3 on the *Irf4* and *Bcl6* genes was validated by ChIP-qPCR analysis. Furthermore, the binding of ROCK2 to both the *Irf4* and *Bcl6* promoters was attenuated by STAT3 silencing as well as by selective ROCK2 inhibitor. Thus, the present study demonstrated previously unidentified evidence that ROCK2-mediated signaling in the cytosol provides a positive feed-forward signal for nuclear ROCK2 to be recruited to the chromatin by STAT3 and potentially regulates TH17/TFH gene transcription.

## Introduction

Rho-associated coiled-coil kinases (ROCKs) play central role in the control of actin cytoskeleton assembly and cellular functions, such as proliferation, adhesion, migration and phagocytosis^1–4^. The two isozymes, ROCK1 and ROCK2, are activated by Rho GTPases and promote actin-myosin mediated contractile force generation via serine-threonine phosphorylation of numerous down-stream targets including myosin light chain (MLC)^5^, myosin binding subunit of myosin phosphatase (MYPT)^6^ and LIM kinase (LIMK)^7^. ROCKs are expressed in both cytoplasmic and nuclear compartments and have been associated with JAK/STAT^8–10^ and p300 signaling pathways in cells^11^. Although ROCK1 and ROCK2 exhibit more than 90% identity within the kinase domain^12^, the functions of these two isozymes are not redundant and depend on the cellular system studied. Using RNA interference, ROCK1 was reported to be critical for stress fiber formation in fibroblasts, whereas ROCK2 controls cortical contractility and phagocytosis^13^. ROCK1 regulates leptin action on body weight homeostasis by activating JAK2^9^, while the ROCK2 protein controls dendritic integrity and memory in the brain^14^. Thus, the activity of each ROCK isozyme must to be evaluated in a cell type- and stimulus-specific manner.

During the immune response, ROCK signaling is critical in the coordination and balancing of T-cell-mediated immune responses, including cellular movement, T-cell receptor (TCR) signaling and the acquisition of the appropriate T-cell effector program^15^. However, only the ROCK2 isozyme was shown to be physiologically activated in CD4^+^ T cells under T helper 17 (TH17) skewing and implicated in development of autoimmunity in mice^16^. In humans, oral administration of the selective ROCK2 inhibitor KD025 to healthy subjects attenuates the ability of T cells to secrete both IL-21 and IL-17 in response to stimulation *ex vivo*^17^. The ROCK2-dependent regulation of TH17 pathway was mediated through STAT3 phosphorylation. Importantly, recent studies demonstrated that targeted inhibition of ROCK2 down-regulates disease progression in MRL/lpr mouse model of lupus and effectively ameliorates clinical pathology in experimental chronic graft-versus-host disease (cGVHD) via inhibition of STAT3 phosphorylation accompanied by a reduced expression of IRF4, RORγt and Bcl6 transcription factors in splenic cells^18,19^.

The decrease in STAT3 phosphorylation occurs within minutes of exposure to the selective ROCK2 inhibitor suggesting that there is a functional crosstalk between ROCK2 and STAT3 signaling pathways in T cells^17^. Here, we report that ROCK1 and ROCK2 isozymes are located in both cytoplasmic and nuclear compartments in human T cells. However, only the ROCK2 protein interacts with phosphorylated STAT3. The ROCK2 kinase activity is required for the formation of JAK-STAT complex leading to optimal STAT3 phosphorylation. Moreover, in the nucleus, ROCK2 and STAT3 chromatin binding highly overlaps and co-localizes to several key genes that control TH17 and T follicular helper (TFH) cell functions, such as *Irf4* and *Bcl6*. The critical role of STAT3 in the recruitment of ROCK2 to chromatin was demonstrated by using siRNA-mediated inhibition of STAT3 as well as by treatment of human T cells with a potent JAK inhibitor.

## Results

### ROCK2, but not ROCK1 interacts with pSTAT3 in both cytoplasmic and nuclear fractions of human T cells

**Figure 1:**
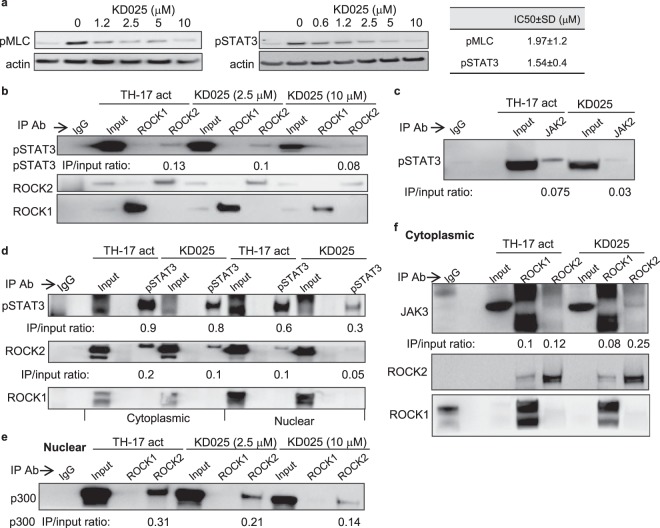
ROCK2 but not ROCK1 interacts with pSTAT3 in both cytoplasmic and nuclear fractions of human T cells stimulated by TH17 skewing conditions. Human peripheral blood CD4^+^ T cells were treated with indicated doses of KD025 and then stimulated under TH17-skewing conditions for 2 hours. (**a**) Total cell extracts were prepared and analyzed by Western blot for pSTAT3 and pMLC expression. (**b**) Co-immunoprecipitation of pSTAT3 by anti-ROCK2 and anti-ROCK1 antibody in the nuclear fraction (**c**) Co-immunoprecipitation of pSTAT3 by anti-JAk2 antibody from total cell lysates of human T cells. (**d**) Co-immunoprecipitation of ROCK2 and ROCK1 with an anti-pSTAT3 antibody in both cytoplasmic and nuclear fractions. Co-immunoprecipitation of p300 (**e**) or JAK3 (**f**) by anti-ROCK2 and anti-ROCK1 antibody in the nuclear (**e**) or cytoplasmic (**f**) fractions. Numbers indicate ratio between the precipitated protein and the input (**e**). One representative of three different experiments is shown. Full-length gels are included in Supplementary Fig. 1.

We previously reported that selective ROCK2 inhibition down-regulates TH17 and TFH cell functions through STAT3-dependent mechanisms in mice and human T cells^17–20^. Using human peripheral blood CD4^+^ T cells stimulated under TH17-skewing conditions we found that a selective ROCK2 inhibitor (KD025) inhibits the phosphorylation of myosin light chain (MLC), one of the ROCK targets, in a concentration-dependent manner with an IC50 of 1.97 ± 1.2 µM. However, KD025-mediated decrease of STAT3 phosphorylation (pSTAT3) occurs at slightly lower IC50 of 1.54 ± 0.4 µM across 4 different experiments (Fig. 1a and Supplementary Fig. 1a), suggesting that STAT3 phosphorylation is more sensitive to selective ROCK2 inhibition in human T cells compared to MLC which is phosphorylated by either ROCK1 or ROCK2 isozymes^5^. In order to further define the molecular mechanism of the exclusive role of ROCK2 in STAT3 activation, we examined the binding partners of ROCK2 in human CD4^+^ T cells. Consistent with findings observed in other cellular experimental systems^11,14,21,22^, both ROCK1 and ROCK2 are detected in cytoplasmic as well as in nuclear fractions of human T cells (Supplementary Fig. 2a). Cell activation and KD025 treatment affected neither the cellular localization nor the protein abundance of ROCK1 and ROCK2 (Supplementary Fig. 2a). We then performed co-immunoprecipitation with lysates of human CD4^+^ T cells stimulated by TH17-skewing conditions in the absence or presence of KD025 for 2 hours. As shown in Fig. 1b and Supplementary Fig. 1b, an anti-ROCK2 antibody can pull down pSTAT3, while an anti-ROCK1 antibody failed to precipitate pSTAT3 in human T cells. Both anti-ROCK1 and anti-ROCK2 antibodies efficiently pulled down the ROCK1 and ROCK2 proteins respectively, and known ROCK targets, such as MLC and MYPT as well (Supplementary Fig. 2b). KD025 treatment diminished the amount of pSTAT3 pulled down by anti-ROCK2 antibody in a concentration-dependent manner. This decrease was partially due to a robust down-regulation of pSTAT3 levels in cells treated with KD025 (Fig. 1a). The formation of pSTAT3/JAK2 complex induced by TH17-skewing was partially abrogated by treatment of cells with the selective ROCK2 inhibitor (Fig. 1c, Supplementary Figs 1c and 2c). In addition, anti-pSTAT3 antibody can specifically precipitate the ROCK2, but not the ROCK1 protein in both cytoplasmic and nuclear fractions, further confirming the specific interaction between ROCK2 and STAT3 in human T cells (Fig. 1d and Supplementary Fig. 1d). Only small fraction of JAK2 interacts with ROCK2, as was demonstrated in pull down experiments by using either ROCK2 or JAK2 antibodies (Supplementary Fig. 2d), suggesting that in the cytoplasmic ROCK2/STAT3/JAK2 complex the interactions occur primarily between ROCK2 and pSTAT3, as well as between pSTAT3 and JAK2. It has been reported that nuclear ROCK2 is present in a large protein complex and partially co-fractionates with p300 in HeLa cells^11^. Also, ROCK2 kinase activity enhances p300 histone acetyltransferase activity and p300-dependent transcription^11^. In human T cells activated by TH17-skewing conditions the ROCK2, but not the ROCK1, isozyme interacts with p300 in the nucleus (Fig. 1e and Supplementary Fig. 1e). The selective ROCK2 inhibitor also diminished the ROCK2/pSTAT3 (Fig. 1b) and ROCK2/p300 (Fig. 1e) interactions in a concentration-dependent manner (numbers in panels indicate the ratio between the precipitated protein and the input). While TH17 skewing induces formation of JAK2/STAT3/ROCK2 complex, STAT5 and JAK3 were almost undetectable in ROCK2 pull down under these conditions in human T cells (Fig. 1f, Supplementary Figs 1f and 2e). However, the presence of KD025 during TH17-skewing promoted the interaction of JAK3 with ROCK2, but not with ROCK1 protein (Fig. 1f), providing a potential molecular mechanism for previously reported increase of STAT5 phosphorylation induced by targeted ROCK2 inhibition both *in vitro* and *in vivo*^17–19^.

### ROCK2 binding is enriched at genomic structures and peaks at transcription start sites of human CD4^+^ T cells

**Figure 2:**
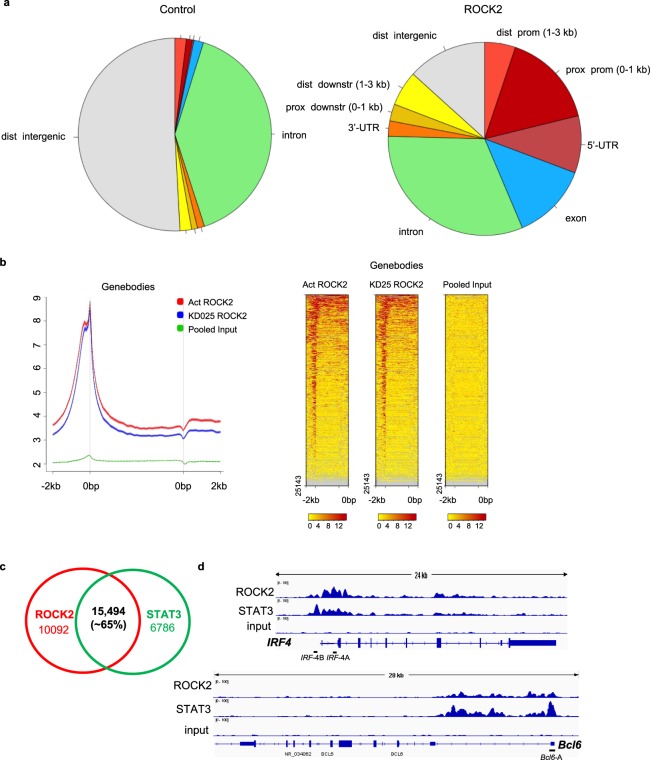
ROCK2 and STAT3 ChIP-seq analysis of human T cell. Human peripheral blood CD4^+^ T cells were stimulated under TH17-skewing conditions for 48 hours; chromatin was purified and proceeded to ChIP-seq analysis with anti-ROCK2 or anti-STAT3 antibodies. (**a**) ROCK2 binding is enriched at genomic structures compared to control. Pie chart of ROCK2 active peaks distribution over control. (**b**) ROCK2 preferentially binds to transcription start sites. Metagene analysis of ROCK2 occupancy on an average gene (left) and Heat map of ChIP-seq reads for ROCK2 occupancy (right). (**c**) Venn diagram with numbers of genomic sites bound by ROCK2 and STAT3. (**d**) An integrative genomics viewer (IGV) browser representation of normalized ChIP-seq reads for ROCK2, STAT3 and input at the *Irf4* and *Bcl6* gene locus. Black bars (*Irf4-*A and *Irf4-*B, *Bcl6-*A) indicate primers used in the ChIP-qPCR analyses in Figs 3, 4.

STAT3 is an essential transcription factor that controls multiple genes involved in regulation of TH17 and TFH cell functions^23–27^. The aforementioned data demonstrated that ROCK2 activity is required for the interaction between JAK2 and pSTAT3 as well as for the ROCK2, but not the ROCK1, protein is associated with p300 in human T cells. To further study the potential role of ROCK2 in regulating gene transcription we performed a ROCK2 ChIP-seq analysis in human CD4^+^ T cells activated by TH17-skewing conditions. Since ROCK2 ChIP or ChIP-seq has not been yet reported, we first validated the anti-ROCK2 antibody before proceeding to a whole genome ChIP-seq (Supplementary Fig. 3a). ChIP-seq analysis reveals that ROCK2 binding is enriched at genomic structures compared to control, with the biggest enrichments at proximal promoter, 5′-UTR and exon regions of these structures (Fig. 2a). This result strongly suggests that the ROCK2 can bind to chromatin. Further analysis of ROCK2 occupancy on an average gene shows that ROCK2 preferentially binds to transcription initiation sites (Fig. 2b). To compare ROCK2 occupancy in the genome to that of STAT3, we also performed STAT3 ChIP-seq analysis of the same sample. Figure 2c shows that about 70% of STAT3 peaks overlap with ROCK2 peaks. The co-occupancy of ROCK2 and STAT3 peaks further indicates that they may cooperate in regulating gene expression. In addition, STAT5 ChIP-seq analysis of the same sample revealed that STAT3 and STAT5 bind to the same consensus sequence and are enriched in the promoter region (Supplementary Fig. 3b). Moreover, STAT3 and STAT5 binding to the chromatin was highly (about 68%) overlapped in human T cells activated by TH17 skewing in consistent with previously reported data in mouse cells (Supplementary Fig. 3c). Treatment of cells with the selective ROCK2 inhibitor KD025 did not change ROCK2 ChIP-seq profiles, as was shown by the similar pie chart data as well as binding enrichment over genomic structures (Supplementary Fig. 3d). Moreover, analysis of active regions shows that ROCK2 binding peaks are at the transcription initiation sites regardless of KD025 treatment (Supplementary Fig. 4a). Detailed genome wide analysis of binding peak intensity found only 10 peaks have increased binding intensity over KD025 treatment, and none of them are related to TH17/TFH cells function (Supplementary Fig. 4b). Among all the analyzed TH17/TFH cell related genes (Supplementary Fig. 4c), STAT3 has one of the highest binding intensity on *Bcl6* gene, consistent with the critical role of STAT3 in regulating *Bcl6* gene expression that was previously reported in both mouse and human cells^27–31^. In fact, ROCK2 has the highest binding intensity on *Irf4* gene compared to other TH17/TFH-related genes (Supplementary Fig. 4c). By using an integrative genomics viewer (IGV) browser representation of normalized ChIP-seq reads for ROCK2, STAT3 and input at the *Irf4* and *Bcl6* gene locus, we found that ROCK2 and STAT3 co-occupied the *Irf4* and *Bcl6 gene* in human T cells activated by TH17-skewing conditions (Fig. 2d).

### Targeted ROCK2 inhibition decreased the binding of ROCK2 to the *Irf4 and Bcl6* promoter

**Figure 3:**
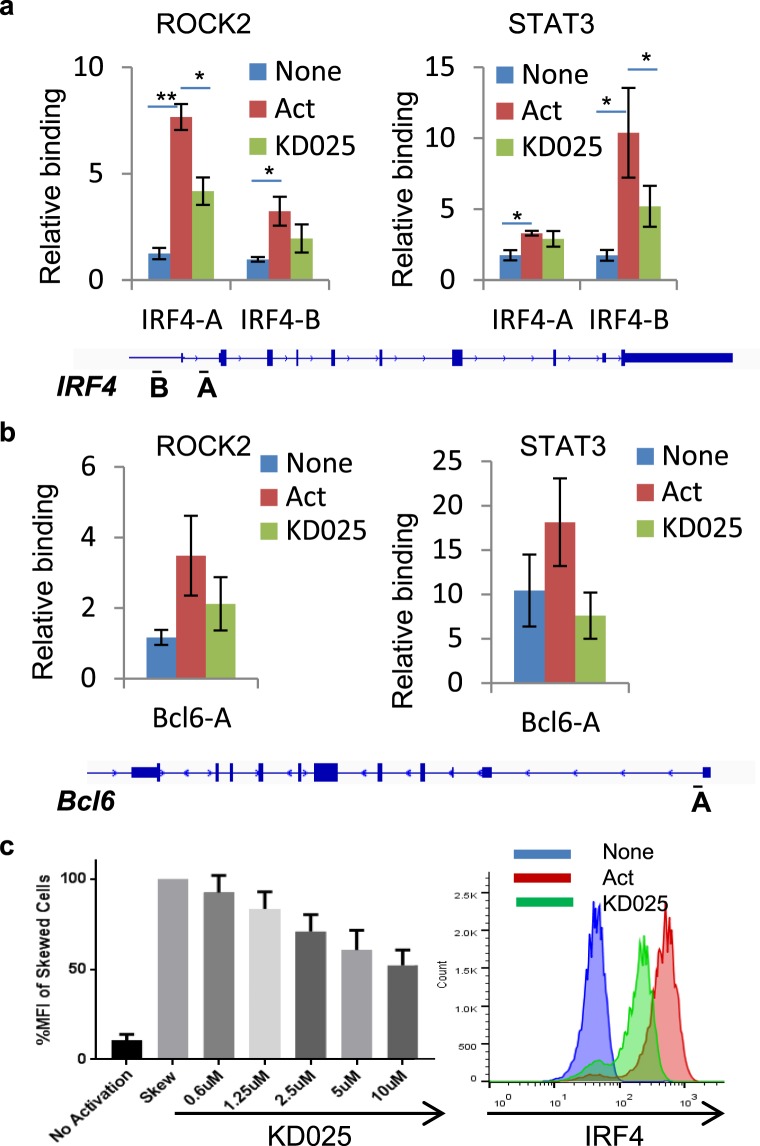
Targeted ROCK2 inhibition decreased the binding of ROCK2 to the *Irf4* and *Bcl6* promoters at Th17-skewing conditions. Human peripheral blood CD4^+^ T cells were stimulated under Th17-skewing conditions for 48 hours in the absence or presence of KD025, chromatin was purified and proceeded to ChIP-qPCR analyses with anti-ROCK2 or anti-STAT3 antibodies (**a**,**b**), or Cells were stained with antibodies to IRF4 and analyzed by Flow Cytometry (**c**,**d**). (**c**) IRF4 expression measured by flow cytometry. Left, Mean Fluorescence Index (MFI) for IRF4 is plotted as percentage of MFI for skewed cells (as 100%). Right, Histogram of the flow cytometry analysis. The average of four different experiments is shown. *p < 0.05; **p < 0.01.

While STAT3 is a well-characterized transcription factor that is known to control both *Irf4* and *Bcl6* genes^31–33^, there is no direct evidence of ROCK2 involvement in the transcriptional regulation of these two genes. Due to the co-occupancy of ROCK2 and STAT3 on the *Irf4* and *Bcl6* promoters and lack of DNA-binding motif in ROCK2^3,15^, we hypothesized that ROCK2 is recruited to *Irf4* and *Bcl6* promoters through its interaction with pSTAT3. To address this question, and to validate the ChIP-seq results, we performed ChIP-qPCR with the human CD4^+^ T cells stimulated by TH17-skewing conditions for 2 days. ROCK2 precipitated with pSTAT3 in both cytoplasmic and nuclear fractions of CD4^+^ T cells after 2 day activation in a similar fashion with the cells at 2 hours (Supplementary Fig. 4d). Then, we performed ChIP-qPCR analyses with anti-ROCK2 or anti-STAT3 antibodies and found that ROCK2 and STAT3 binding to *Irf4* and *Bcl6* promoters are significantly enriched during TH17 skewing compared to unstimulated cells (Fig. 3a,b). Although ROCK2 inhibition did not affect the ROCK2 binding to the whole genome (Fig. 2a and Supplementary Fig. 4a), KD025 treatment decreased the ROCK2 and STAT3 occupancy on the *Irf4* and *Bcl6* promoters by 50% (Fig. 3a,b). We observed that binding of ROCK2 is higher at the *Irf4-A* site versus the *Irf4-B* site (approximately 1 kb upstream of TSS). However, binding of STAT3 is higher at the *Irf-B* site, and very weak at the *Irf4-A* site. This was consistent with our observation from ChIP-seq results (Fig. 2d). The recruitment of ROCK2 and STAT3 to the *Irf4* promoter correlates with a significant increase in the expression level of IRF4 in human CD4^+^ T cells activated by TH17 skewing and it was reduced by KD025 treatment in a concentration-dependent manner (Fig. 3c).

### STAT3 mediates ROCK2 binding to chromatin

**Figure 4:**
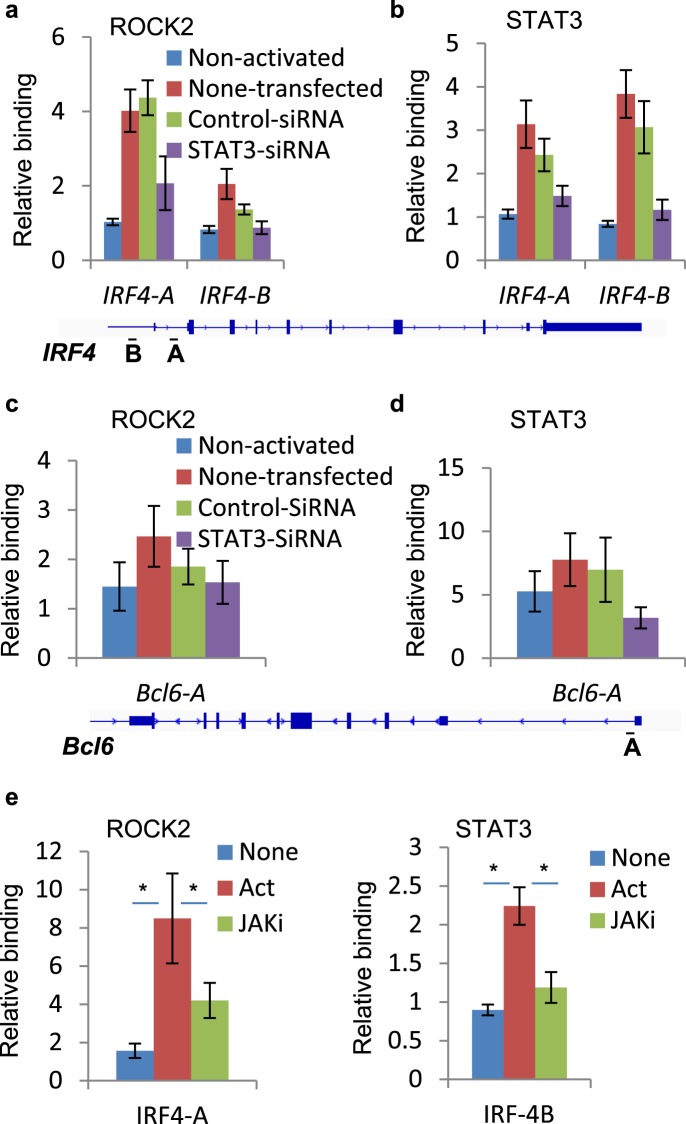
Knockdown of STAT3 decreases binding of ROCK2 to *Irf4* promoter. Human peripheral blood CD4^+^ T cells were transfected with control or STAT3 siRNA, before stimulated under Th17-skewing conditions for 48 hours (**a–d**) or treated with a JAK inhibitor, Baricitinib while being stimulated under Th17-skewing conditions for 48 hours (**e**). Chromatin was purified and proceeds to ChIP-qPCR analysis with anti-ROCK2 (**a**,**c**,**e**) or anti-STAT3 antibodies (**b**,**d**,**e**). The average of three or four different experiments is shown. *p < 0.05.

To further confirm the critical role of STAT3 in the recruitment of ROCK2 to the *Irf4* and *Bcl6* promoters, we used STAT3-specific small interfering RNA (siRNA) that reduced STAT3 expression by more than 90% in human CD4^+^ T cells (Supplementary Fig. 4e). Moreover, ChIP-qPCR analysis revealed that knock down of STAT3 robustly decreased the binding of ROCK2 to the *Irf4* promoter, especially to the *Irf4-A* site, compared to non-transfected or cells transfected with control siRNAs (Fig. 4a). The binding of STAT3 at the *Irf4* promoter (Fig. 4b) and the *Bcl6* promoter (Fig. 4d) was completely abrogated in cells transfected with STAT3 siRNAs. Although ROCK2 binding to the *Bcl6* promoter was partially reduced in STAT3-silenced cells, the results were inconclusive due to the weaker binding of ROCK2 and the effect of control siRNA compared to non-transfected cells we observed in some experiments (Fig. 4c). In addition, STAT3 phosphorylation as well as the binding of both ROCK2 and STAT3 to the *Irf4* promoter was robustly decreased by treatment of cells with a JAK inhibitor, Baricitinib (Supplementary Fig. 4f and e). Thus, these data further confirm the essential role of JAK in regulation of TH17-induced STAT3 phosphorylation and ROCK2 recruitment to the chromatin in human CD4^+^ T cells activated by TH17-skewing conditions.

## Discussion

The small GTPase RhoA activates its down-stream targets ROCK1 and ROCK2 which regulate cellular functions through the control of the actin cytoskeleton assembly and cellular contraction^3,4^. Although these two isozymes share more than 90% homology within their kinase domain and have some common substrates, it has been demonstrated by several groups that the cellular functions of ROCK1 and ROCK2 are not redundant^12–14,34^. By using siRNA specific knockdowns, ROCK inhibitors and ROCK2 haploinsufficient mice it was shown that the ROCK2, but not ROCK1 protein is implicated in regulation of TH17 and TFH pathways^16–19^. However, both the molecular mechanism as well as the specific contribution of ROCK2 to the development of autoimmunity is still enigmatic. Here we report that ROCK2, but not ROCK1, interacts with STAT3 in primary human T cells activated by TH17-skewing. More specifically ROCK2 interacts with pSTAT3 in the cytoplasm (Figs 1b,d), followed by the recruitment of both ROCK2 and STAT3 to the TH17/TFH related gene promoters in the nucleus (Fig. 2 and Supplementary Fig. 4c). It was previously reported that TH17-skewing selectively activates ROCK2 in both mice and humans^16,35^. Consistent with that, the present study shows that TH17-induced ROCK2 kinase activity as demonstrated by increased MLC phosphorylation was down-regulated by the selective ROCK2 inhibitor KD025 that was in correlation with a decrease in STAT3 phosphorylation in cells (Fig. 1a). Importantly, KD025 partially abolished the TH17-induced formation of JAK2/STAT3/ROCK2 complex (Fig. 1b–d) which is required for optimal activation of STAT3 phosphorylation, suggesting that intact ROCK2 kinase activity is essential for the complex formation as well as STAT3 phosphorylation. Interestingly, only small fraction of JAK2 was detected in pull down experiments with anti-ROCK2 antibody compared to pSTAT3 (Supplementary Fig. 2d), indicating that in the JAK2/STAT3/ROCK2 complex the interaction occurs primarily between ROCK2 and pSTAT3, as well as between pSTAT3 and JAK2. We also observed that inhibition of ROCK2 kinase simultaneously promotes interaction of ROCK2 with JAK3 (Fig. 1f) which could be responsible for up-regulation of STAT5 phosphorylation in T cells as was previously reported in both *in vitro* and *in vivo* experiments with KD025^17–19^. It should be noted however that STAT5 was undetectable in both ROCK1 and ROCK2 pull-downs in either untreated or KD025-treated cells (Supplementary Fig. 2e), suggesting an indirect effect of ROCK2 inhibition on the induction of STAT5 phosphorylation in T cells. Further studies are required to define the role of ROCK2 kinase activity in the regulation of ROCK2 and JAK3 interaction and STAT5 phosphorylation.

The functional crosstalk between ROCK and JAK/STAT pathways has been documented in other cell types. It was shown that only the ROCK1 isozyme interacts with JAK2 which increases downstream activation of STAT3 in neurons stimulated by leptin. This suggests a potential role of ROCK1 in regulation of energy homeostasis^9^. In cancer cells and fibroblasts, ROCK activation induced STAT3 phosphorylation in a time frame similar to that for increased MLC phosphorylation^8,10^. Although intact ROCK kinase as well as JAK kinase activity is required for ROCK-induced STAT3 phosphorylation^10^, it is not clear if ROCK1 and ROCK2 isozymes contribute equally to the regulation of STAT3 phosphorylation in cancer cells and fibroblasts. The present study demonstrated that only the ROCK2 protein interacts and regulates STAT3 activation (Fig. 1b,d), whereas both ROCK1 and ROCK2 isozymes can bind canonical ROCK targets, such as MLC and MYPT in primary human T cells (Supplementary Fig. 2b). Furthermore, we propose that the ROCK2 kinase activity modulates the scaffolding function of ROCK2 in the cytosol; thus promoting the assembly of JAK2/STAT3/ROCK2 complex, while negatively regulating the interaction of JAK3 and ROCK2 in T cells activated by TH17-skewing conditions (Fig. 1f).

Besides its roles in cytoskeleton reorganization in the cytosol, ROCK2 is localized in the nucleus of cancer cells, epithelial cells and neurons^11,14,21,36^. Despite the fact that both ROCK isozymes are present in the nucleus of primary human T cells (Supplementary Fig. 2a), only the ROCK2 co-immunoprecipitated with pSTAT3 as well as p300 (Fig. 1e), a key regulator of chromatin acetylation and promoter activity^37,38^. The interaction between ROCK2 and p300 has been previously reported in Hela cells^11^, suggesting involvement of ROCK2 in regulating transcription. Here we provide evidence that ROCK2 is recruited to chromatin, preferentially at transcription initiation sites, which highly overlapped, genome-wide, with STAT3 occupancy and which co-localized to several key genes involved in regulation of the TH17 and TFH cell function, such as *Irf4* and *Bcl6* (Fig. 2). ROCK2 cannot bind to chromatin directly due to lack of DNA-binding motif^3,15^. We hypothesized and confirmed by STAT3-specific silencing that STAT3 mediates the recruitment of ROCK2 to the *Irf4* and *Bcl6* promoters and therefore might play a critical role in ROCK2-controlled regulation of TH17/TFH gene expression (Fig. 4). In addition, the association of nuclear ROCK2 with p300, which acetylates chromatin to open up specific transcription sites, can make chromatin more accessible for basal transcription machinery and facilitate the initiation of transcription during TH17-skewing activation. The hypothesis is also supported by evidence that phosphorylation of p300 by ROCK2 leads to enhanced p300 histone acetyltransferase activity^11^. However, the mechanism of how the ROCK2-p300 interaction affects chromatin states needs further investigation by using ChIP-seq with epigenetic markers during TH17-skewing activation. ROCK2 can directly phosphorylate IRF4 and regulate its DNA binding ability in mouse T cells^16^, providing additional evidence of ROCK2 involvement in transcriptional regulation. Furthermore, selective ROCK2 inhibition decreased the ROCK2 and STAT3 occupancy on the *Irf4* and *Bcl6* promoters followed by a decrease in IRF4 (Fig. 3) and Bcl6 protein levels^19^. Thus, nuclear ROCK2 appears to be instrumental in regulation of TH17 and TFH gene expression in human T cells via STAT3-mediated mechanism.

While we are beginning to explore the therapeutic potential of targeted ROCK2 inhibition in the clinic, several reports demonstrated that KD025 effectively ameliorates animal models of autoimmunity, including collagen-induced arthritis^17^ and Mrl/lpr lupus^19^ in mice as well as reversed the pathology of the autoimmune-like syndrome, chronic graft-versus-host disease (cGVHD)^18^. Notably, in both animal models and patient-derived cells the decrease in disease pathology was associated with robust down-regulation of pSTAT3, IRF4 and Bcl6 levels, while STAT5 phosphorylation was increased^17–19,39^. These data underlie the functional consequences of crosstalk between ROCK2 and STAT3 signaling pathways in both cytoplasm and nucleus of T cells and its contribution to regulation of the TH17/TFH gene expression during auto-reactive immune responses. Furthermore, the finding from a phase 2, open-label clinical study (NCT02317627 at ClinicalTrials.gov) demonstrated that oral administration of KD025 reduced clinical scores in patients with psoriasis vulgaris, normalized skin pathology and down-regulated ROCK2, pSTAT3 and IRF4 levels further confirming the molecular mechanism of targeted ROCK2 inhibition^20^.

**Figure 5:**
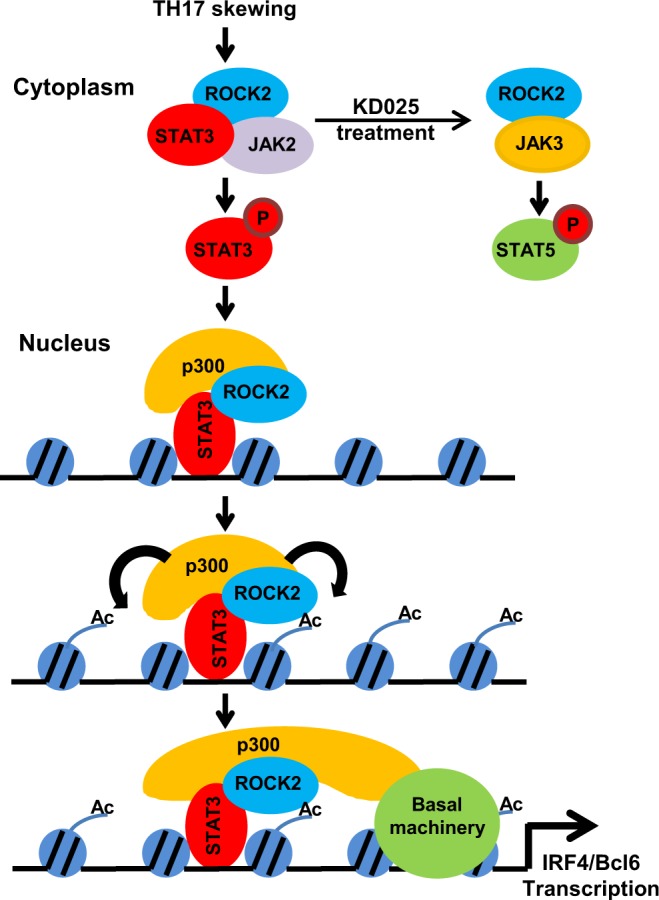
A proposed model for the mechanism of ROCK2 in regulating TH17/TFH gene expression. Under TH17-skewing conditions, ROCK2 specifically interacts with STAT3 in the cytosol, and ROCK2 kinase activity is required for the formation of JAK2/STAT3 complex and optimal STAT3 phosphorylation in human T cells. In addition, targeted ROCK2 inhibition promotes interaction of ROCK2 with JAK3 which could be responsible for up-regulation of STAT5 phosphorylation. In the nucleus, ROCK2 interacts with phosphorylated STAT3 as well as p300, and is recruited to the *Irf4* and *Bcl6* promoters by STAT3. Thus, ROCK2 cooperates with STAT3 in both cytoplasmic and nuclear compartments in T cells stimulated by TH17-skewing conditions.

In summary, our findings demonstrate the specific role of ROCK2 in the regulation of STAT3 phosphorylation and following TH17/TFH gene expression in T cells. Our hypothesis (Fig. 5) suggests that the ROCK2 interacts with STAT3 in the cytosol, and ROCK2 kinase activity is required for the formation of JAK2/STAT3 complex, optimal STAT3 phosphorylation and translocation to the nucleus. These early signaling events provide a positive feed-forward signal for nuclear ROCK2 to be recruited to the chromatin by STAT3, and specifically to the *Irf4* and *Bcl6* promoters in human T cells. In addition, nuclear ROCK2 associates with p300 which facilitates the initiation of gene transcription, possibly by regulation of chromatin acetylation. All together these data suggest that ROCK2 can amplify the pro-inflammatory signaling cascade by synergizing with STAT3 in both cytoplasmic and nuclear compartments in T cells stimulated by TH17-skewing conditions.

## Materials and Methods

### Cell purification and activation

Total CD4^+^ T cells were purified from the peripheral blood of healthy human donors between the ages of 16 and 75 years (New York Blood Center, NY, NY) as was previously described^17^. Peripheral blood units used for this study were collected under approved ethic committee/institutional review board (IRB) protocol (New York Blood Center) and informed consent was obtained from all subjects. All methods were carried out in accordance with relevant guidance and regulations. Total CD4^+^ T cells were activated toward TH17 cells with immobilized anti-CD3 mAb (5 µg/ml) and anti-CD28 mAbs (5 µg/ml) (eBioscience, San Diego, CA) with IL-1β (50 ng/ml) and TGF-β (5 ng/ml) (R&D Systems Inc, Minneapolis, MN). The selective ROCK2 inhibitor KD025 (formerly Slx-2119) was dissolved in DMSO. In addition to ROCK1, KD025 was found to have no significant activity against 300 intracellular kinases and surface receptors^17^.

### Co-immunoprecipitation (co-IP) assay

Cytoplasmic and nuclear fractions were prepared using NE-PER nuclear and cytoplasmic extraction reagents (Thermo scientific #78835) and subjected to western blot analysis or to co-IP. Co-IP specific antibodies (anti-ROCK1 (sc-6055; sc-374388) and anti-ROCK2 (sc-398519) from Santa Cruz Biotechnology; anti-ROCK2 (R8653) from Sigma; anti-pSTAT3 (Tyr705) (#4113) from Cell Signaling Technology) were immobilized on Dynalbeads ProteinG (Thermo Fisher Scientific Inc.) as instructed by manufacturer. JAK2 (D2E12) conjugated Sepharose beads were purchased from Cell Singling Technology (#4089). For co-IP, cytoplasmic or nuclear extracts were incubated overnight with the antibody-conjugated dynalbeads or sepharose beads. The beads were then washed with buffer (20 mM Tris-Hcl (pH 7.5), 20% glycerol, 0.1 mM EDTA, 300 mM NaCl, 0.5 mM DTT, 0.5 mM PMSF, 0.1% NP40) five times, and Tris/EDTA (TE) once. The bound proteins were eluted by incubating at 70 °C for 10 minutes in TE/1%SDS buffer and subjected to western blot analysis. Other antibodies used for western blot analysis are anti-phospho-MLC (Invitrogen PA5-17727); anti-P300 (SC-48343) and anti-JAK3 (SC-6932) from Santa Cruz Biotechnology; anti-MLC (#85053), anti-MYPT (#26345), anti-STAT5 (#94205) from Cell Signaling Technology.

### Flow cytometry

Human CD4^+^ T cells were incubated with PBS in the presence of a viability dye (Viability Dye eFluor 780 eBioscience 65-0865-18) before being washed with flow cytometry staining buffer (eBioscience 00-4222-26), then fixed and permeabilized using a Transcription Factor Staining Buffer Set (eBioscience 00-5523-00). Cells were then incubated with Anti-Human/Mouse IRF4 PE-Cyanine7 (eBioscience 25-9858-82) and washed. Cells were analyzed on a Millipore Guava Flow Cytometer 8HT and the machine was compensated using appropriate single color controls. Analysis of flow cytometry data files was performed using FlowJo version 10.0.7.

### Chromatin Immunoprecipitation (ChIP) Assays and ChIP-seq analysis

Peripheral blood CD4^+^ T cells were activated by anti-CD3/28 mAbs, IL-1β and TGF-β in the absence or presence of 10 µM KD025. Cells were harvested at 48 hour for ChIP assays as previously described or for ChIP-seq analysis. ChIP-seq analyses were performed by Active Motif FactorPath^TM^ ChIP-seq service. Antibodies used for ChIP-seq: anti-STAT3 (Santa Cruz biotechnology, sc-482), anti-STAT5 (Santa Cruz biotechnology, sc-835) and anti-ROCK2 (Sigma R8653).

### RNA interference

Mixture of four ON-TARGETplus SMARTpool siRNAs specific to STAT3 (L-003544-00) and control siRNA (D-001810-10-20) were purchased from Dharmacon (Thermo Fisher Scientific Inc., Waltham, MA). Transfections of freshly purified T cells were performed using the human T cell Nucleofector kit (Amaxa Biosystems, Lonza, Basel, Switzerland). Transfection efficiency was controlled by evaluating STAT3 level using Western Blot analysis after 48 hours.

### Statistics

Data were analyzed by two-tailed Student t-test by using the GraphPad Prism software.

## Electronic supplementary material

Supplementary materials
