# Supplementary material for: ROCK2, but not ROCK1 interacts with phosphorylated STAT3 and co-occupies TH17/TFH gene promoters in TH17-activated human T cells
Source: Sci Rep. 2018 Nov 9;8:16636. doi: 10.1038/s41598-018-35109-9 (PMC6226480; doi:10.1038/s41598-018-35109-9)
Supplement: Supplementary file 1 — Supplementary materials [file 41598_2018_35109_MOESM1_ESM.pdf]

# ROCK2, but not ROCK1 interacts with phosphorylated STAT3 and co-occupies TH17/TFH gene promoters in TH17-activated human T cells

Wei Chen[1], Melanie S. Nyuydzefe[1], Jonathan M. Weiss[1], Jingya Zhang[1], Samuel D. Waksal[1, 2,] and Alexandra Zanin-Zhorov[1]*

[1]Kadmon Corporation, LLC, New York, NY 10016

[2]Current Weill Cornell Medicine, New York, NY 10021

*Correspondence: Alexandra.Zanin-Zhorov@kadmon.com; Kadmon Corporation, LLC, 450 East 29th street, New York, NY 10016. Tel: 212-308-6000

*Running title: ROCK2 interacts with STAT3 in human T cells*

# Supplementary Figure 1

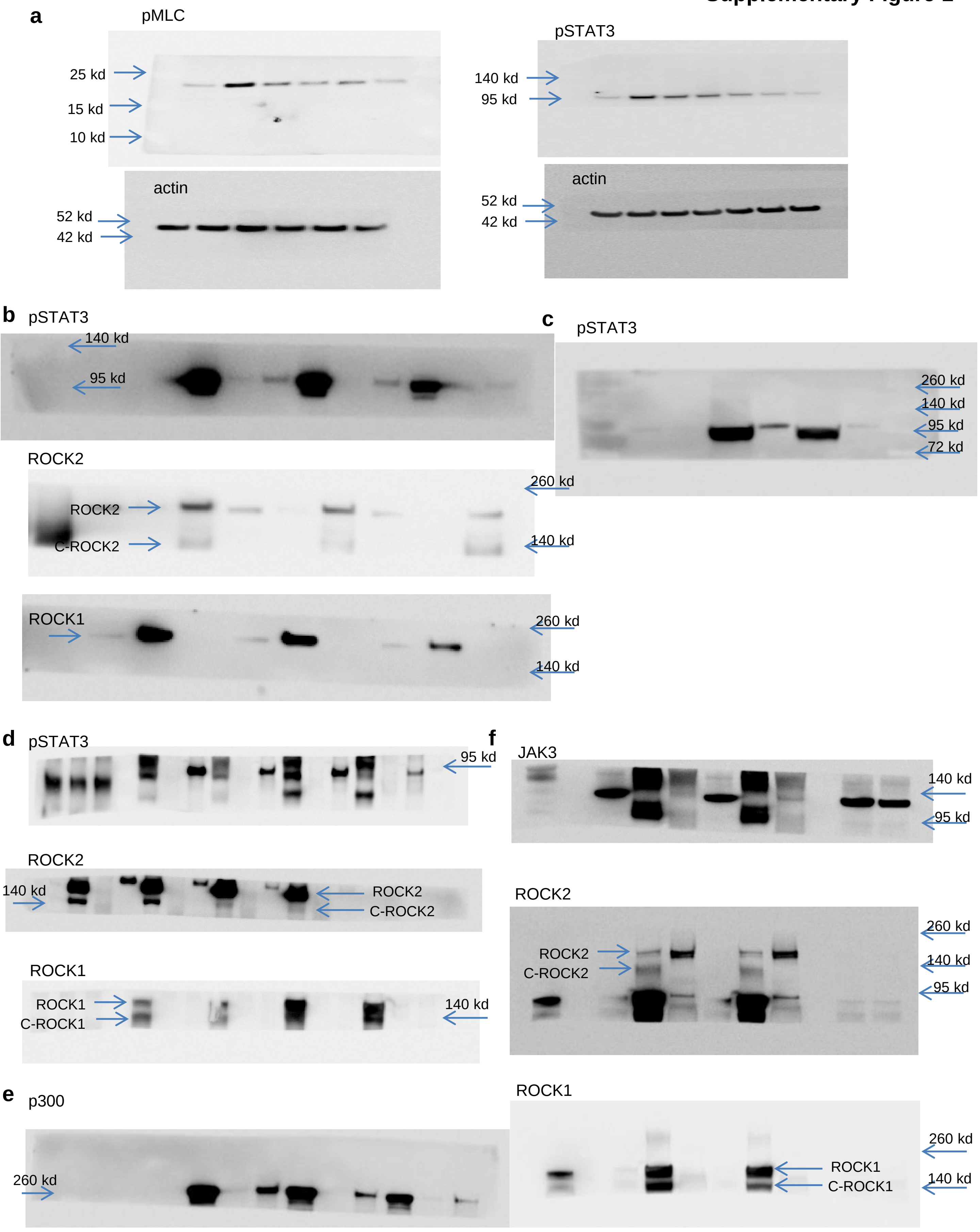

## Supplementary Figure 2

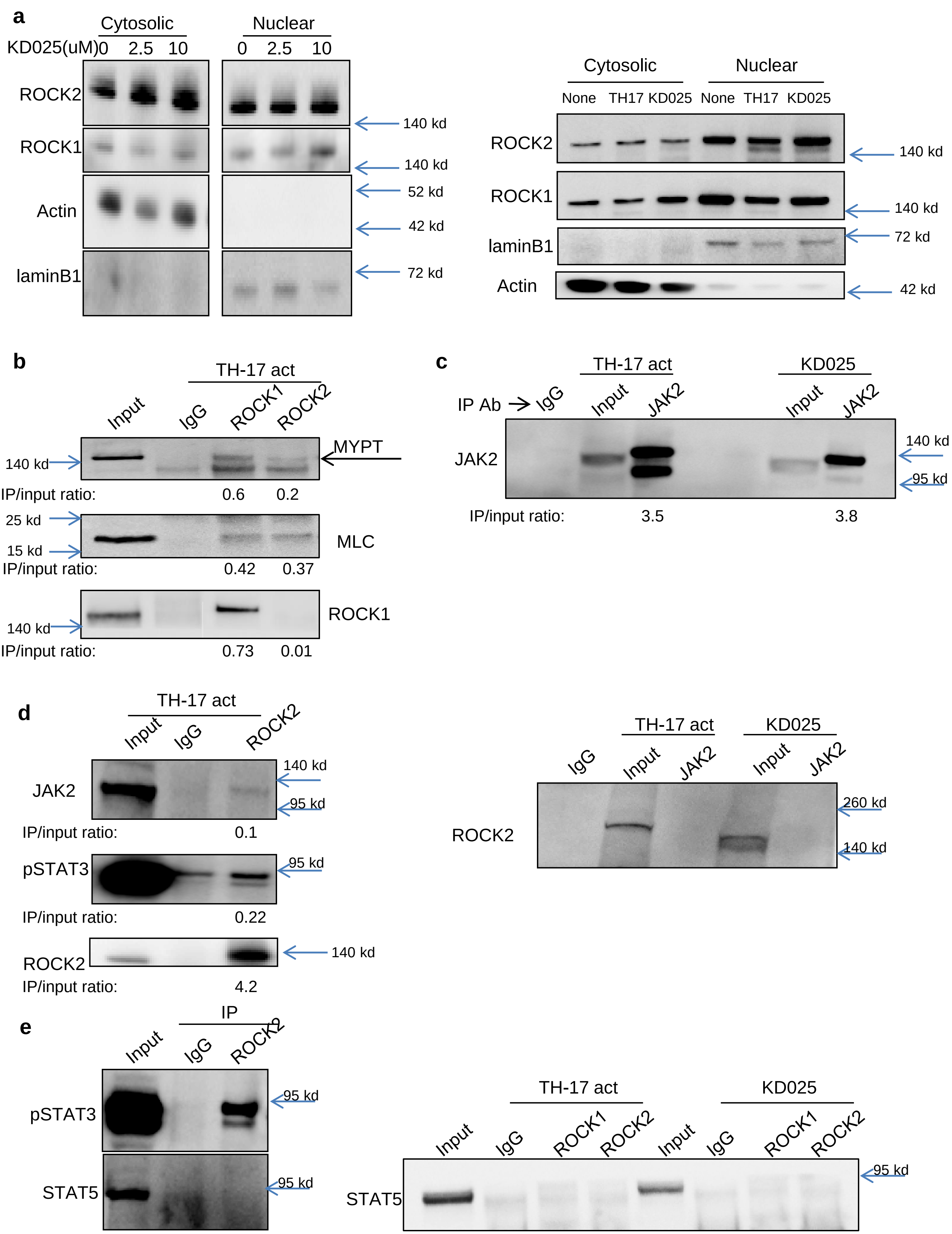

## Supplementary Figure 3

a

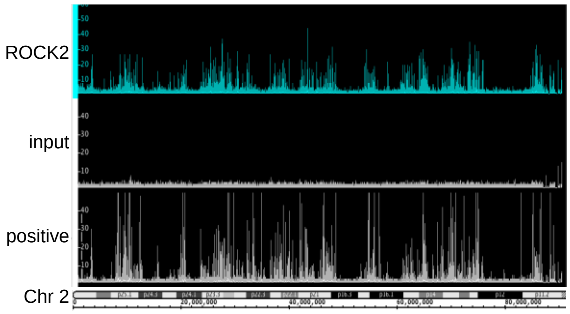


b

STAT3

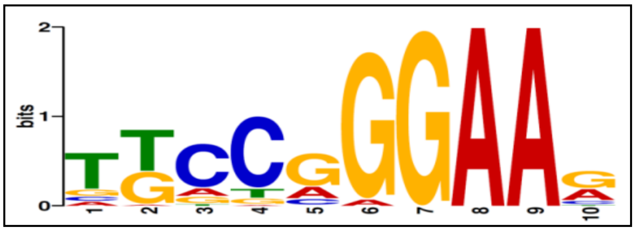


STAT5

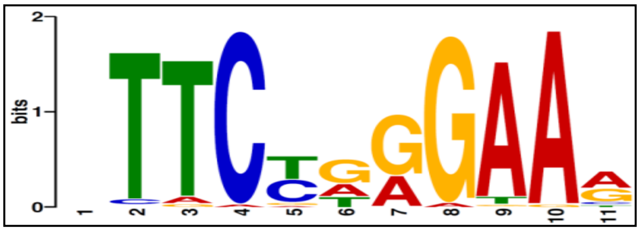


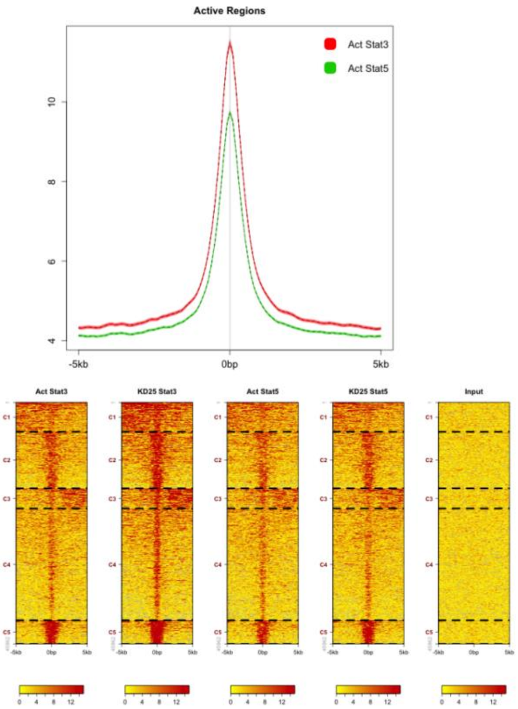


c

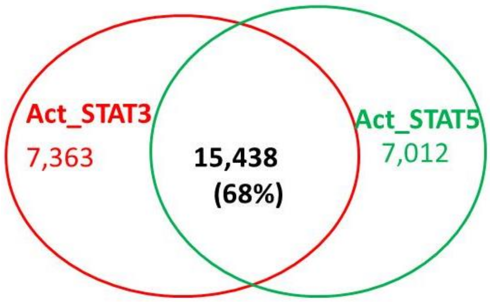


d

KD025 ROCK2

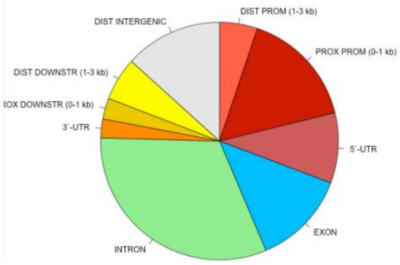


| Genomic regions | Control | Act ROCK2 | KD025 ROCK2 |
|---|---|---|---|
| Dist prom (1-3 kb) | 1.94% | 5.65% | 5.75% |
| Prox prom (0-1 kb) | 1.03% | 14.85% | 17.81% |
| 5’-UTR | 0.25% | 8.47% | 10.65% |
| Exon | 1.61% | 13.1% | 14.34% |
| Intron | 40.6% | 38.2% | 35.42% |
| 3’-UTR | 1.19% | 3.06% | 2.86% |
| Prox downstr (0-1 kb) | 0.99% | 3.2% | 3.17% |
| Dist downstr (1-3 kb) | 1.96% | 6.51% | 6.53% |
| Dist intergenic | 51.27% | 16.78% | 14.79% |

# Supplementary Figure 4

a

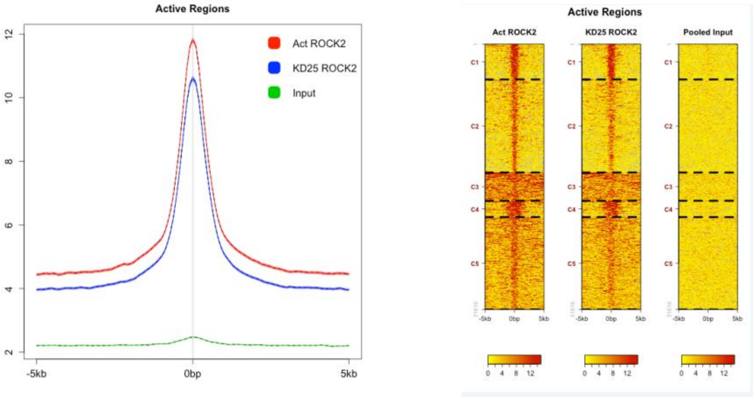


b

| | Act ROCK2_increased | KD025 ROCK2_increased |
|---|---|---|
| **Number of peaks** | 348 | 75 |
| **Number of peak with value >50** | 0 | 10 |

c

| | Genes bound by ROCK2 | | Genes bound by STAT3 | |
|---|---|---|---|---|
| Gene List | Dist to Start | ROCK2 Peak Value | Dist to Start | Stat3 Peak Value |
| BCL6 | 957 | 41 | 775 | 106 |
| PRDM1 | -60 | 16 | 514 | 22 |
| PRDM1 | 1,478 | 16 | 11,989 | 87 |
| PRDM1 | 12,236 | 15 | 14,674 | 9 |
| IRF4 | 1,026 | 77 | 662 | 79 |
| RORC | 14,749 | 24 | 6968, 15653, -13565 | 139 |
| CXCR5, BCL9L | 713, 26359 | 18 | -403 | 31 |
| ICOS | -1,727 | 15 | 69 | 23 |
| PDCD1, C2orf85 | -10883, 55 | 23 | 23 | -400 |
| BATF | -321 | 22 | 870 | 113 |
| MAF | -89 | 39 | -179 | 54 |
| STAT3 | 134 | 59 | 219 | 270 |
| TXLNA | 663 | 35 | 780 | 163 |
| TOB1 | -2462, -22 | 37 | -2459, -25 | 50 |

d

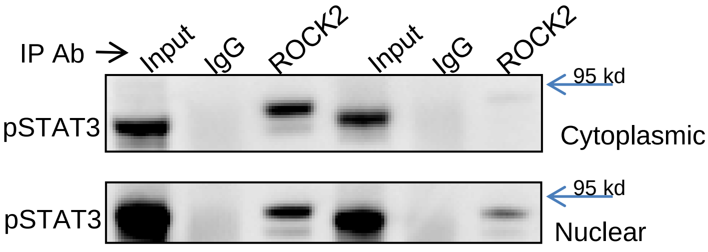


e

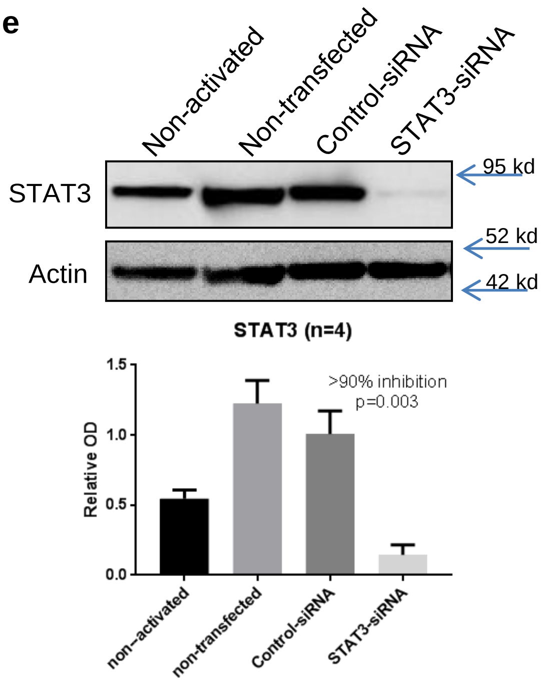


f

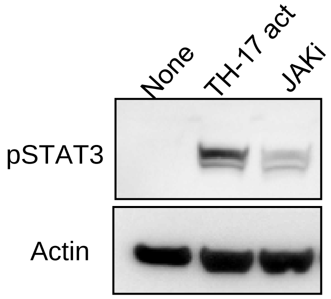

**Figure 1. Full-length gels for Figure 1**

**Figure 2. Both ROCK1 and ROCK2 exist in the cytoplasmic as well as the nuclear fraction of human CD4$^+$ T cells.** Human peripheral blood CD4$^+$ T cells were stimulated under Th17-skewing conditions for 2 hours, then nuclear and cytoplasmic fractions were purified and subjected to western blot analyses. (a) Actin and LaminB1 served as quality controls for cytoplasmic and nuclear fractions respectively. (b) Both anti-ROCK1 and anti-ROCK2 antibodies can co-precipitates MYPT and MLC. (c) Anti-JAK2 antibody specifically pulls down JAK2. pSTAT3 (d), but not STAT5 (e), co-immunoprecipitated with ROCK2. (d) Weak JAK2 being detected by anti-ROCK2 co-immunoprecipitation, but no detectable ROCK2 by anti-JAK2 co-immunoprecipitation (d). Numbers indicate ratio between the precipitated protein and the input.

**Figure 3. ROCK2, STAT3 and STAT5 are co-localized on chromatin in human T cells.** (a) A screen shot of ROCK2 peaks, together with input and a positive antibody at Chromosome 2 from ROCK2 antibody ChIP-seq validation assay. (b) STAT3 and STAT5 occupancy on an average gene peak at transcription start region; STAT3 and STAT5 binding motif. (c) Venn diagram with numbers of genomic sites bound by STAT3 and STAT5. (d) Pie chart of ROCK2 active peaks distribution in KD025-treated cells over control.

**Figure 4. STAT3 mediates ROCK2 recruitment to the chromatin.** (a) ROCK2 binding peaks at transcription start site regardless KD025 treatment. (b) Number of peaks changed after KD025 treatment shows KD025 does not alter genome wide ROCK2 binding profile. (c) List of TH17/TFH cell related genes with high ROCK2 and STAT3 peaks analyzed by ChIP-seq. (d) ROCK2 interacts with pSTAT3 in human CD4$^+$ T cells stimulated by TH17 skewing conditions for 48 hours. (e) STAT3 siRNA robustly down-regulates the expression of STAT3, a representative Western blot and quantification from four independent experiments is shown. (f) Phosphorylation of STAT3 is decreased by treatment of cells with a JAK inhibitor, Baricitinib.
